# Trainable Bilingual Synaptic Functions in Bio-enabled Synaptic Transistors

**DOI:** 10.1021/acsnano.3c04113

**Published:** 2023-09-18

**Authors:** Moon Jong Han, Vladimir V. Tsukruk

**Affiliations:** † Department of Electronic Engineering, Gachon University, Seongnam 13120, Republic of Korea; ‡ School of Materials Science and Engineering, Georgia Institute of Technology, Atlanta, Georgia 30332, United States

**Keywords:** brain-inspired computing, photonic cellulose nanocrystals, bio-organic field-effect transistors, neuromorphic behaviors, optoelectronic synaptic devices

## Abstract

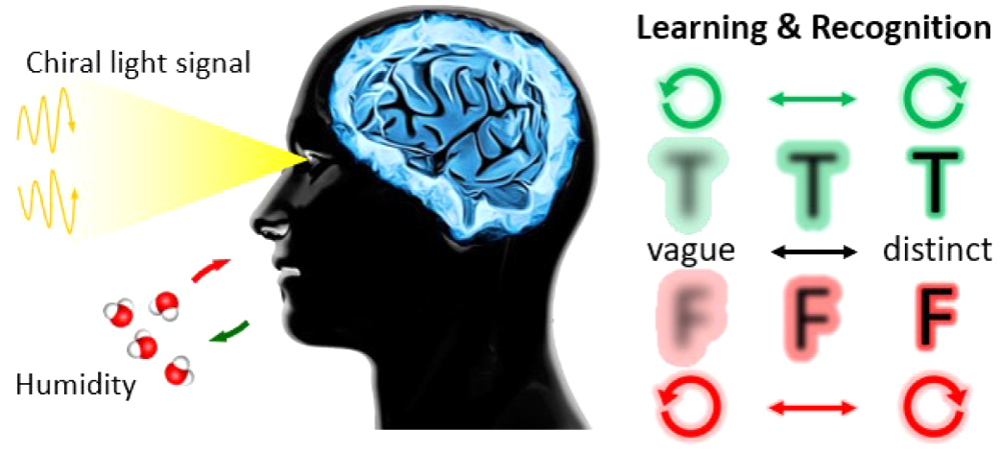

The signal transmission of the nervous system is regulated by neurotransmitters. Depending on the type of neurotransmitter released by presynaptic neurons, neuron cells can either be excited or inhibited. Maintaining a balance between excitatory and inhibitory synaptic responses is crucial for the nervous system’s versatility, elasticity, and ability to perform parallel computing. On the way to mimic the brain’s versatility and plasticity traits, creating a preprogrammed balance between excitatory and inhibitory responses is required. Despite substantial efforts to investigate the balancing of the nervous system, a complex circuit configuration has been suggested to simulate the interaction between excitatory and inhibitory synapses. As a meaningful approach, an optoelectronic synapse for balancing the excitatory and inhibitory responses assisted by light mediation is proposed here by deploying humidity-sensitive chiral nematic phases of known polysaccharide cellulose nanocrystals. The environment-induced pitch tuning changes the polarization of the helicoidal organization, affording different hysteresis effects with the subsequent excitatory and inhibitory nonvolatile behavior in the bio-electrolyte-gated transistors. By applying voltage pulses combined with stimulation of chiral light, the artificial optoelectronic synapse tunes not only synaptic functions but also learning pathways and color recognition. These multifunctional bio-based synaptic field-effect transistors exhibit potential for enhanced parallel neuromorphic computing and robot vision technology.

## Introduction

The human brain is highly effective in computing, using parallel and event-driven architectures to process complicated data.^1,2^ It employs four trillion synapses and neurons to complete calculations,^3−5^ which are based on excitatory and inhibitory neurons.^6^ This provides the brain’s neural network with excitatory and inhibitory synapses of several functionalities.^7^ The versatility of the nervous system originated from the balancing process of excitatory and inhibitory synaptic inputs.^8,9^ The presynaptic stimuli of neurons regulate the performance of postsynaptic neurons, such as excitation or suppression, which can be applied to innovative intelligent platforms for signal recognition, processing, and identification.^10^ For instance, a presynaptic stimulus makes electronic outputs of excitatory postsynaptic current (EPSC), inhibitory postsynaptic current (IPSC), paired-pulse acceleration (PPF), potentiation, or depression within postsynaptic neurons.^11,12^ The excitation/suppression ratio of neurons is controlled by the balance of excitation–suppression, which directly affects the stability and circuitry functionalities.^13^ The complementary excitability and the inhibitory input increase the dynamic range of sensitivity of the nervous system, allowing for a broad function range.^14,15^ This enables the brain to manage complicated data processing in numerous forms of visual, auditory, and tactile inputs.^16−19^

The synaptic behavior of the ventral tegmental area neurons’ single axon terminal is considered reconfigurable and bilingual due to the ability to produce both excitatory and inhibitory neurotransmitters.^20^ Despite the numerous synaptic transistors that have been demonstrated, they usually exhibited monosynaptic properties.^21,22^ Most synaptic transistors are based on organic semiconductors and ion gel dielectrics, and their operational mechanism is based upon temporary charge transport and dynamics, making it challenging to achieve both excitatory and inhibitory synaptic behavior.^23,24^

A great deal of effort has been put into understanding the interactive balance between excitatory and inhibitory properties of the nervous system to imitate the efficient human brain functioning.^25−27^ For instance, wired transistor arrays were created to copy intercalated neuronal architectures.^28,29^ Nevertheless, due to the need for a complicated arrangement of multiple connections, it was restricted to balance demonstration between excitation and suppression.^30^ As an alternative, multifunctional perovskite quantum dots,^31^ carbon nanotubes,^32^ and 2D materials^33,34^ have been suggested to realize the complementary nervous system. By providing photoelectronic characteristics and anisotropy characteristics, the outputs of the 2D material devices can be activated by various inputs. For instance, the synaptic reaction can be simulated by using both electrical and optical inputs simultaneously.^35^ With multifunctionality equivalent to high integration, both excitatory and inhibitory synapse features can be mimicked.^36^ However, it is challenging to manage the simultaneous balance of both excitation and suppression with the same voltage pulses.^37^ Potential resolution is the manipulation of signs of the input pulses that make processing more cumbersome.^38−40^

Apart from their synaptic functions, photonic synapses have been studied to apply to artificial vision systems, which have suggested unique advantages for light-induced activation. For example, a heterojunction photonic synapse that could imitate the mechanical aperture device for adaptive optical perception allowed for neuromorphic behavior and exhibiting visual memory behavior.^41^ Especially, a photonic synapse that achieved similar functions with different materials was also reported.^42^ Unfortunately, the photonic synapse changes could not distinguish diverse colors in a single device system because the optical functions of functional layers were limited in their abilities to recognize the colored pattern in the human visual system.

Herein, we suggest integrated bio-synaptic transistors with balanced inhibitory and excitatory functions by facilitating the humidity-responsive bio-electrolyte layer. In this system, the active layer of chiral CNC/PEG/NaCl composite films and optoelectrically triggered semiconducting conjugated channels are combined in response to the polarization state of light and relative humidity (RH) conditions. Within active bio-electrolyte-gated transistors (BEGTs), the injection of charges from gate electrodes or mobile ions in the electrolyte material or the slow polarization of permanent dipoles modulates the backward current during transfer curves. As the concentration of water molecules is further increased under higher humidity conditions, the additional water molecules can act like traps for the charge carriers at the accumulation layer, leading to a decrease in backward current. The synaptic functions of the artificial synapses are tuned by the humidity conditions and hysteresis phenomena of EGTs. The synaptic transistors exhibit balancing characteristics of the excitatory or inhibitory response of the devices, which are regulated by the same value of input pulses. The plasticity of the artificial synapse element could be controlled by the balance of the excitatory and inhibitory inputs. Additionally, the transistors emulated the EPSC, IPSC, and PPF characteristics and revealed the possibility of achieving long-term potentiation (LTP) and long-term depression (LTD). The artificial synapse transistor suggested here emulates excitatory and inhibitory synaptic functions, which could affect the memory functions of the brain. Moreover, by applying different wavelengths and circular polarization of light, a photonic synaptic element can not only distinguish two distinct colors (red and green) but also facilitate polarization recognition to mimic the environmental adaptation and selective memory functions of the advanced human visual system for future robotic vision.

## Results and Discussion

### Fabrication of Artificial Synapse Based on Active Electrolyte Layers

**Figure 1:**
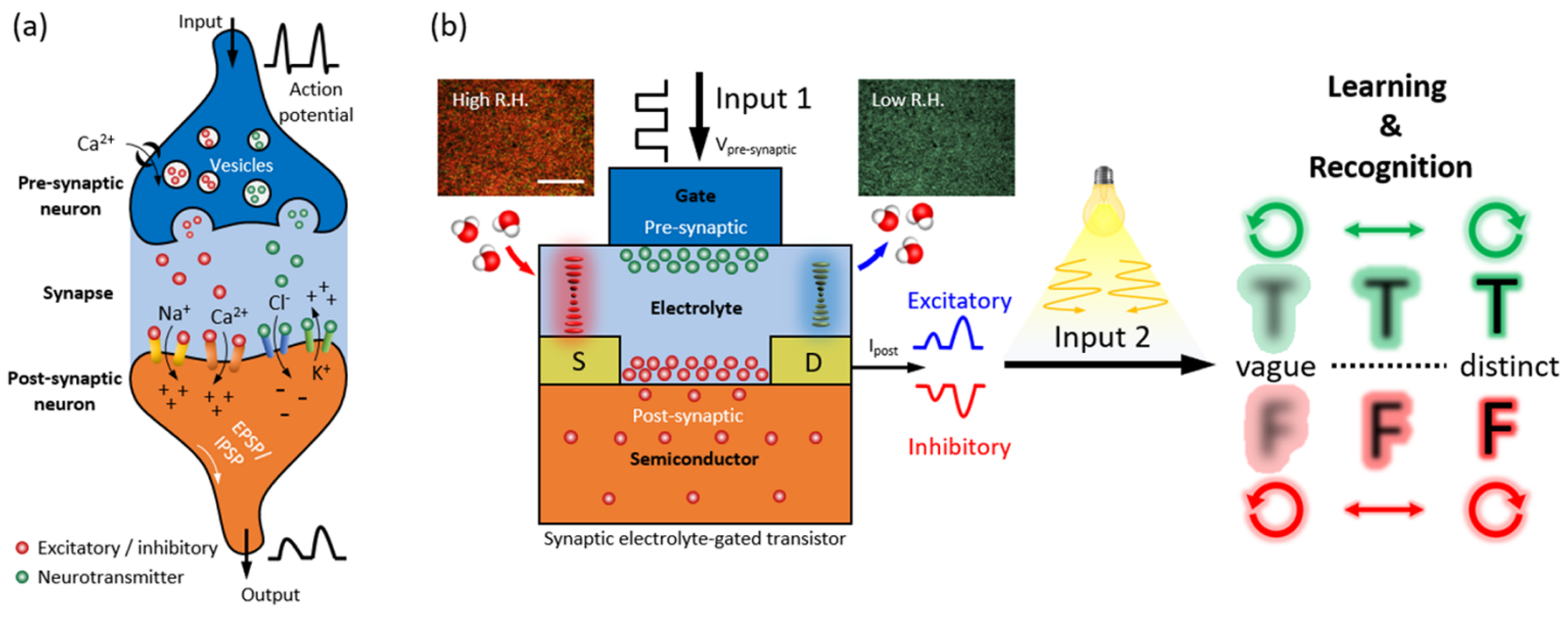
Schematic of (a) a biological synaptic system^45^ and (b) the advanced electrolyte-gated transistor (EGT) triggered by voltage pulses (input 1) and chiral light irradiation (input 2), emulating learning and recognition of neuromorphic computing (inset scale bar: 200 μm). Depending on humidity, the synaptic EGT modulates the chiral pitch of the active electrolyte layer and slows polarization of CNC composites, tuning optoelectrical behavior directly in the systems. By adjustment of the polarization of the incident light (input 2), the advanced system distinguishes the letters differently.

In the nervous brain structures, an electrical synapse usually possesses an ∼30 nm gap^43^ that connects two neurons and is able to process presynaptic inputs to trigger the postsynaptic outputs (Figure 1a).^44,45^ This connection between neurons, known as the synaptic weight (W), is determined by the concentrations of ions, such as Na^+^, Ca^2+^, and K^+^, which are activated by presynaptic action potentials and control the release of neurotransmitters. This synaptic weight allows neurons to be linked, transmitting electrical or chemical signals and influencing the spiking behavior of nearby neurons, thus leading to neuronal growth. Changes in synaptic strength are referred to as synaptic plasticity, which is dependent on either or both sides of the synapse.^46^ This plasticity is usually divided into short-term plasticity (STP) and long-term plasticity (LTP). STP is a temporary modification of synaptic strength after stimulation, which can last from milliseconds to a few minutes, while LTP is a lasting alteration of synaptic strength that can last from hours to years. STP is necessary for short-term memory (STM) and helps synapses to do important computations in neural circuits such as transmission, encoding, and filtering of neuronal signals.^47^ On the other hand, LTP is needed for storing processed information, i.e., long-term memory (LTM), and is thought to be the foundation of learning and memory. STP can be transformed into LTP after sufficient training or persistent neuronal activities. The nervous system relies on the combination of excitatory and inhibitory synapses to enable computational and learning processes.^48,49^ The postsynaptic currents (PSCs) are determined by the ratio of excitatory and inhibitory synaptic inputs.^7^

As is known, an electrolyte can act as an ionic conductor as well as an electronic insulator dielectric.^50^ An electrolyte could be either in a liquid or in a solid state, having ions (anions and cations) displaced in opposite charges at the electrolyte/electrode interfaces in response to an electric field. Specifically, the impermeable EGT with the channel current modulated by the gate voltage (input 1) is governed by a capacitive field effect at the channel/electrolyte interface. Due to the ultrathin “electrical double layers” (EDLs, 1.0 nm) formed at the gate/electrolyte and electrolyte/semiconductor interfaces, EGTs are often known as electrical double-layer transistors.^51^ In comparison to a thickness-dependent dielectric layer, a high parallel plate capacitance (∼1–10 μF/cm^2^) or volumetric capacitance (∼500 μF/cm^2^) is utilized to connect the gate and the channel.^52^ This characteristic gives synaptic EGTs the capability to modify conductance at extremely low voltages (∼mV), making them a desirable alternative in energy-efficient neuromorphic circuits.

Here, we exploit the cellulose nanocrystal (CNC) EGTs to implement the artificial synapses (Figure 1b and Figure S1). By deploying the CNC/PEG/NaCl electrolyte into the element of an EGT, it allows exploring how the presence of water molecules tunes the volatility, thus balancing the excitatory and inhibitory functions. Note that the absorption and desorption of water molecules cause expansion or shrinkage of chiral pitch, resulting in a reddish and greenish color appearance, corresponding to high and low RH conditions in the system, respectively.^53,54^ The photonic input 2 is additionally introduced to emulate the process by which the human brain obtains information from the outside world through the visual system. For example, in the demonstrated neuromorphic device, the green and red letters can be accurately identified by modulating the polarization state of light and humidity conditions (Figure 1b).

### Electrical Characterization of CNC Composite-Based FETs Responding to Relative Humidity

**Figure 2:**
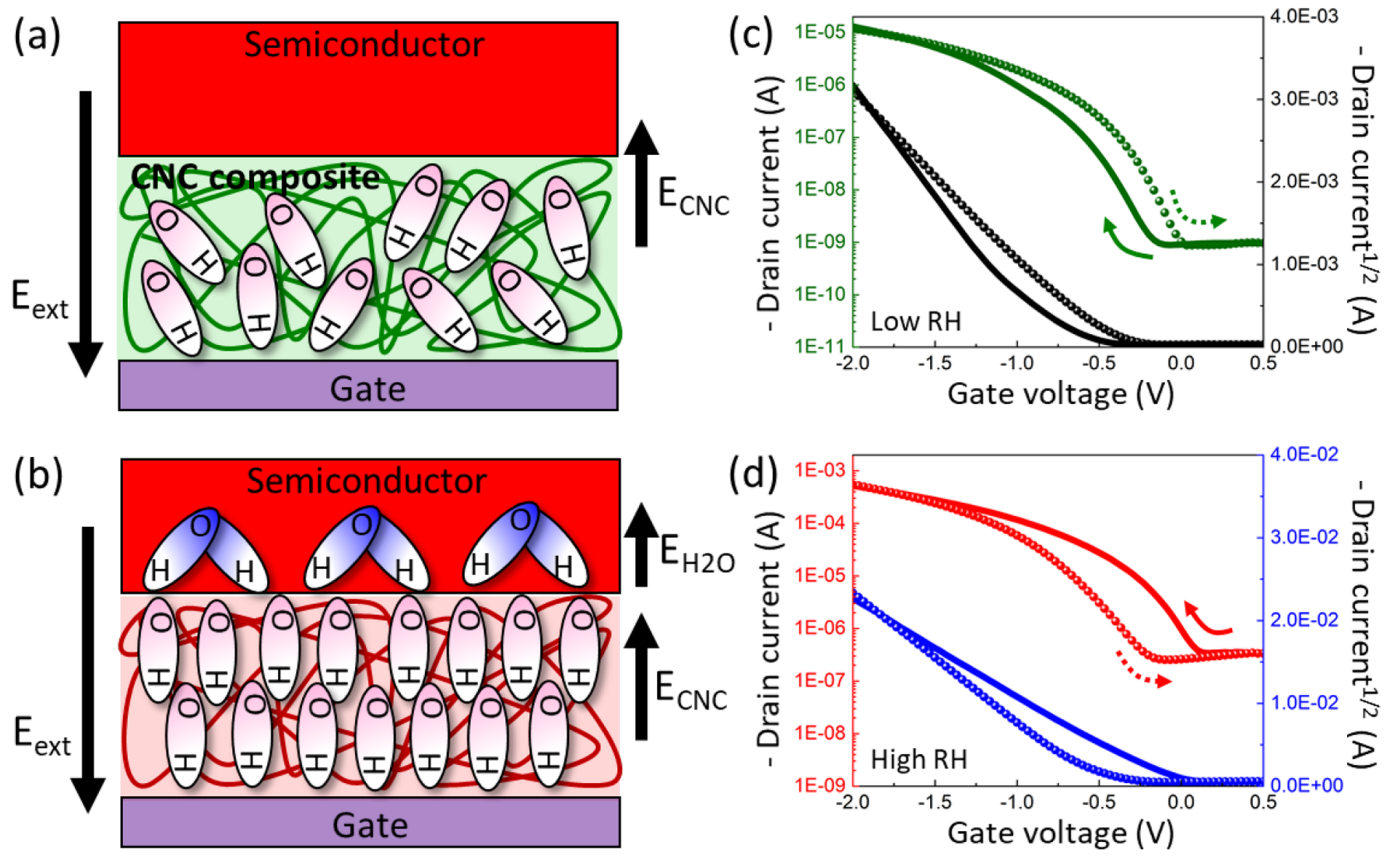
Tunable electrical hysteresis of active electrolyte layer-based synaptic devices. Partial polarization of dipoles of −OH groups within the CNC composite layers under an applied gate voltage at (a) 42% and (b) 92% RH conditions. At relatively low humidity, the slow polarization of permanent dipoles in the PEG molecule’s dielectrics induces a higher current of backward-sweeping current compared with that of forward sweeping. Enhanced polarization of the −OH groups and absorbed water molecules in the semiconductor/electrolyte layer interfaces trigger the charge trappings and lower currents of backward-sweeping compared to forward-sweeping current.

To examine the behavior of CNC-based synaptic EGTs under normal environmental conditions, a systematic study was conducted to analyze their response and stability under controlled-humidity conditions (Figure 2a–d). The transfer characteristics curves of the p-type semiconductor channel of PBTTT-C14 presented in Figure 2c,d and Figure S2 show measurements taken at various RH levels ranging from 25 to 92% RH. It was discovered that as humidity increased, the hysteresis decreased at 67% RH but then increased as the humidity rose to 92% RH. The *I*_DS_ value during backward sweeping was observed to be higher than that during forward sweeping up to 67% RH, which was attributed to the anomalous hysteresis effect caused by charges injected from the gate electrodes, mobile ions in the dielectric material, or the slow polarization of permanent dipoles in the PEG molecule’s dielectrics (Figure 2a).^55,56^

However, above 67% RH, the forward-sweeping current exceeded the backward-sweeping current, as is typical in most cases, and is usually attributed to charge trapping at the interface.^57^ As water molecules accumulate, the −OH groups become polarized and generate more charges in the accumulation region. The adsorbed water molecules at the interface of the semiconducting layer also increase in density due to the applied gate field, which polarizes the adsorbed water molecules. This enhancement enhances the polarization of permanent dipoles in the PEG layer of the CNC composites, causing the −OH groups to polarize more rapidly under high humidity conditions (92% RH) than under relatively low humidity conditions (42% RH) (Figure 2b).^58^ Therefore, the hysteresis caused by slow polarization of −OH groups decreases as RH increases, and at 67% RH, almost no hysteresis was observed. This may be due to the saturation of adsorbed molecules at the interface at high humidity, where the water dipole-generated field is strong enough to completely saturate the polarization of −OH groups in the PEG molecules.^59^ Under high humidity conditions, the device exhibited higher *I*_DS_ values compared to those for conditions below 67% RH due to the concentration of water molecules, which produced an extra gate field by the shift of protons from the absorbed water molecules in CNC composite layers, inducing excess holes in the channel. For the n-type semiconductor channel of ITIC-F, the transfer characteristics followed the same output phenomena. As the humidity increased, the hysteresis initially decreased at 67% RH, but then it rose again as the humidity continued to increase up to 92% RH.

For the EGT system, the hysteresis of conductance has shown that it is related to the relaxation time of potential distribution while the relaxation time of distribution is sensitive to the sweeping rate.^60,61^ As such, the capacitive gating from oriented dipoles competes with charge trapping, and a slow gate sweeping rate could increase the hysteresis from capacitive gating and decrease the hysteresis from charge trapping (Figure S3). As the capacitive gating effect is dominant due to the existence of dipoles, clockwise hysteresis occurs at a low sweeping rate.

Under low humidity conditions (Figure S3a–c), the enhancement of the local electrical field near the semiconducting layers induces more majority carriers by capacitive gating, and then the carrier density in the semiconducting layer is effectively increased. A clockwise hysteresis is finally observed. However, for high humidity conditions (Figure S3d–f), the excessively absorbed water molecules, which have close contact with the semiconducting layer, decrease the charge carrier mobility as leakage current increases because the excessively generated protons in the CNC complex layer find a favorable path to move toward the interface due to the gate electric field and contribution to the higher leakage current.^62,63^ As the charge trapping by the water molecules within the CNC composite/semiconductor layers is dominant compared with the existence of dipoles, anticlockwise hysteresis increases at a higher sweeping rate.

However, there still remain limitations to quantitatively determining the exact traps by the various factors and the traps themselves originated from various intrinsic and extrinsic sources.^64^ Additionally, it is difficult to individually control the influencing factors and to confirm the hidden factors.^65^ Therefore, there is a need to study further the corresponding trap sources in the future.

### Synaptic Behavior of CNC Composite-Based EGT

**Figure 3:**
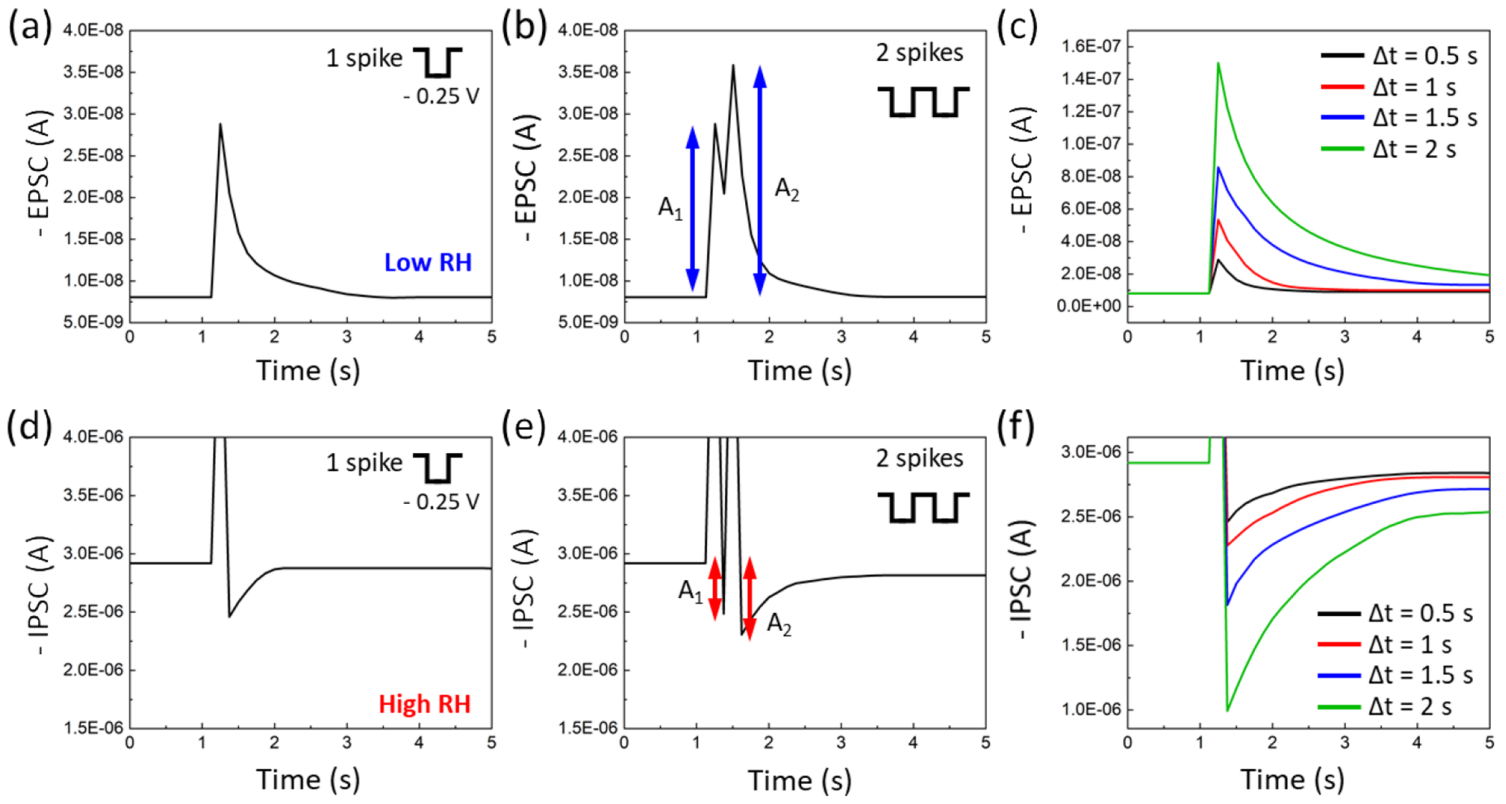
Synaptic behavior of the active electrolyte-gated transistors. EPSC triggered by (a) single and (b) double spikes of −0.25 V, 0.5 s. (c) EPSC retention curves by different pulse duration times such as 0.5, 1, 1.5, and 2 s. IPSC triggered by (d) single and (e) double spikes of −0.25 V, 0.5 s. (f) IPSC retention curves by different pulse duration times such as 0.5, 1, 1.5, and 2 s.

An excitatory postsynaptic current (EPSC) of −6.43 nA was produced by a single presynaptic spike (−0.25 V, 0.5 s) at low RH conditions, 42% RH, which decayed to a resting current of about −8 nA within a few seconds due to slow polarization of permanent dipoles in the electrolyte (as shown in Figure 3a). In biological synapses, a process called paired-pulse facilitation (PPF) occurs when a pair of pulses are applied in quick succession, resulting in an increase in postsynaptic signals.^66,67^ This phenomenon is a common property of biological synapses and reduces the time between pulses (Δ*t*) and amplifies the postsynaptic potentiation, leading to short-term synaptic enhancement such as temporal postsynaptic plasticity in sensory and motor nervous systems. In the artificial synapse, when paired pulses with Δ*t* = 0.5 s were applied (Figure 3b), the second EPSC peak (A_2_) was amplified by approximately 30% compared to the first EPSC peak (A_1_). This is because the second pulse causes an additional accumulation of permanent dipoles of PEG before the accumulation during the first pulse has completely diffused away. As a result, the EPSC increased correspondingly, as the induced charges at the electrolyte surfaces increased. The first sentence states that the PPF (A_2_/A_1_) decreases as the Δ*t* increases, which leads to a decrease in the number of residual anions accumulated by the first peak (Figure S4). This response is familiar to that observed in biological synapses. The suggested BEGT operates reliably under different presynaptic spike forms while exhibiting various synaptic properties such as PPF (A_2_/A_1_), spike-voltage-dependent plasticity (Figure S5), spike-number-dependent plasticity, and spike-frequency-dependent plasticity. The magnitude of EPSC increases with the voltage of the presynaptic spike (−0.05 to −0.25 V in increments of −0.05 V).

Figure 3c shows EPSC retention with different duration times of 0.5, 1, 1.5, and 2 s. Following the voltage pulse to the gate electrode, the excitatory and inhibitory postsynaptic currents instantaneously reached the peak and gradually decreased to their initial value upon the voltage pulse removal for low humidity conditions.

For high RH conditions, the higher water content acts as a trap for charge carriers in the accumulation layer, resulting in an inhibitory postsynaptic current (IPSC) of −2.49 μA by a single presynaptic spike of −0.25 V with 0.5 s time duration (Figure 3d). The paired pulses with Δ*t* = 0.5 s were applied, and the second IPSC peak (A_2_) was amplified by approximately 1.32 times compared to the first IPSC peak (A_1_) (Figure S4). For the IPSC retention measurements, different pulse duration times were manipulated such as 0.5, 1, 1.5, and 2 s (Figure 3f). The IPSC decreases instantaneously to each peak, followed by an increase to its initial value upon voltage pulse removal.

To confirm the multibit data storage characterization of the CNC composite-gated transistors, consecutive V_G_ was introduced under V_D_ = −2 V. After five consecutive sweeps of negative V_G_ were swept from 0 to −0.25 V (0 V → –0.25 V → 0 V), *I*_D_ showed an increase and decrease under low (Figure S6a) and high humidity (Figure S6b) conditions, respectively, with distinguishable states for 60 s. The results exhibited the multilevel data storage capability of the EGTs.

### Electrical Artificial Synaptic Characterization of CNC Composite-EGT

**Figure 4:**
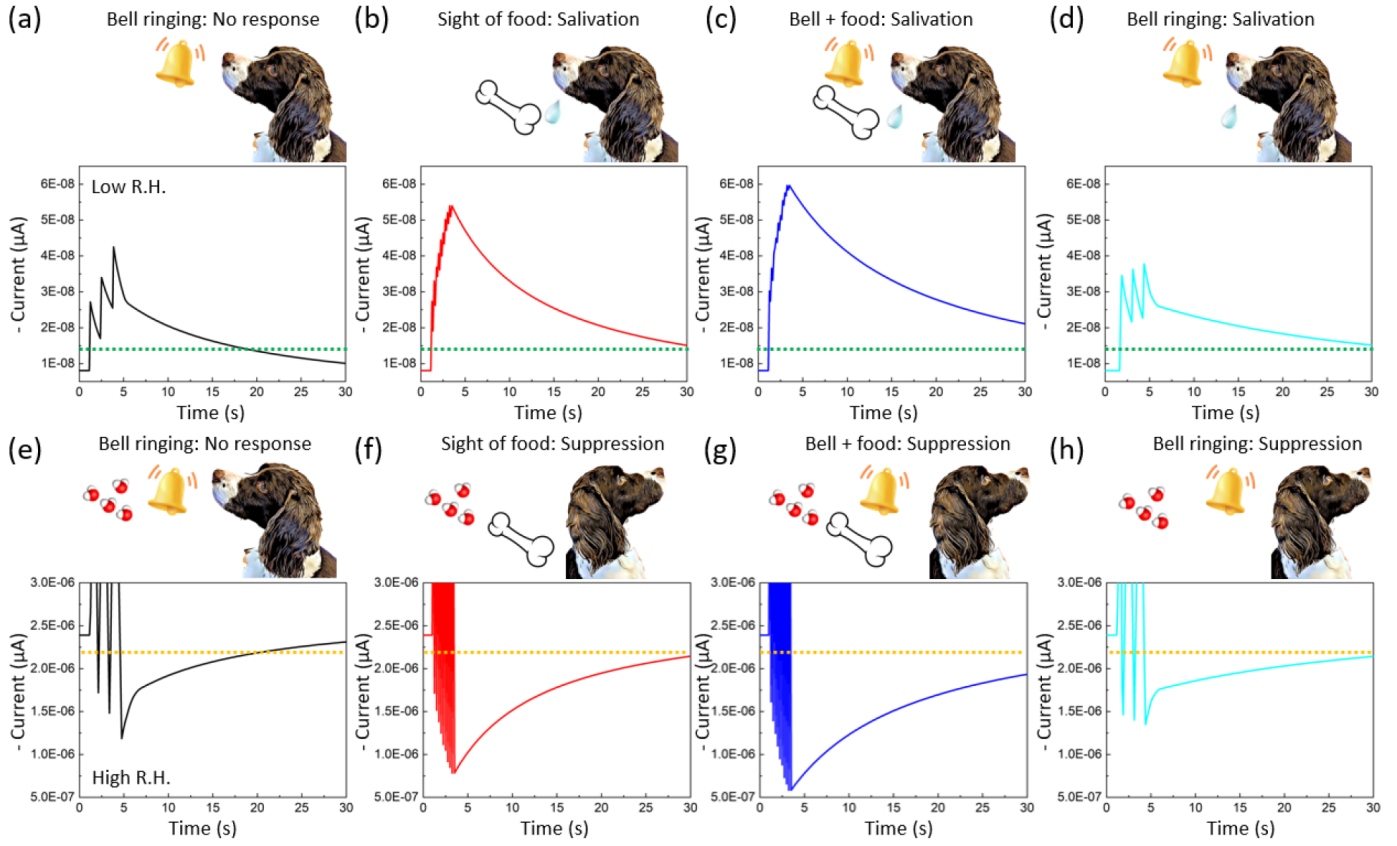
Pavlov’s learning at different humidity conditions At low humidity (42% RH), (a) three −0.25 V electric pulse signals (at *T*_p_ = 200 ms, Δ*t* = 2 s) were introduced to the G1 electrodes to simulate the “bell ringing”. A small output of Δ*W*_peak_ is compared with the defined threshold, where green dotted lines correspond to the absence of salivary responses. (b) Ten −0.25 V electric pulses (at *T*_p_ = 200 ms, Δ*t* = 200 ms) were applied to the G2 electrode to simulate the “sight of food”, corresponding to the salivary responses, where Δ*W*_peak_ is higher than the threshold. (c) Simultaneous three and ten electric pulses at G1 and G2 electrodes are applied for the “training”. (d) After training, a larger Δ*W*_peak_ than the salivary response is definitely produced, even with three −0.25 V pulses, corresponding to bell ringing. For high humidity conditions (92% RH), (e–h) the inhibitory behavior of the synaptic device generates depression of appetite with the bell-ringing process after the training process.

As the next step, we deployed CNC composite-EGT to simulate classical conditioning based on Pavlov’s learning rule, which has been emulated in memristors and transistors.^34,68^ For example, classical conditioning using a single memristor was emulated by pairing positive and negative bias voltages as unconditioned and conditioned stimuli. In the present work, we simulated classical conditioning in multigate devices depending on humidity to emulate excitatory and inhibitory behavior (Figure 4).

For both low and high RH conditions, we used three −0.25 V pulse signals to simulate the “bell ringing” and ten −0.25 V pulse signals to simulate the “sight of food”, resulting in a significant increase or decrease in Δ*W*_peak_ for output neurons corresponding to salivation (Figure 4a–d) and suppression (Figure 4e–h) of appetite, respectively. During training, input signals were applied simultaneously to the G1 and G2 electrodes to cause large changes in the Δ*W*_peak_ of the device. After training, the Δ*W*_peak_ of the device increased significantly even when the input signal was only applied to the G2 electrode, similar to a puppy salivating or depression of appetite when the bell rings.

During the first stage, the G2 input signal (which corresponds to the unconditioned stimulus of the bell ringing) caused a slight change in Δ*W*_peak_ from the output neurons, while the G1 input signal (which corresponds to the conditioned stimulus of the sight of food) resulted in a significant change in Δ*W*_peak_ for the output neurons (which corresponds to salivation). In the training stage, input signals were applied simultaneously to the G1 and G2 electrodes, leading to large changes in the Δ*W*_peak_ of the device. After training, even when the input signal was applied only to the G2 electrode, Δ*W*_peak_ of the device could still be significantly increased, which is similar to a puppy salivating when the bell rings.

Figure 4a–d displays the result of the simulated learning under low humidity conditions. The third graph shows the training process. We set a threshold of 14.5 nA depicted by the green dotted line to determine whether our multiterminal synaptic device learned to associate the food signal with the ringing signal. Initially (Figure 4a), three −0.25 V pulse signals (at *T*_p_ = 0.5 s and Δ*t* = 2 s) were applied to the G2 electrode to simulate the “bell ringing”, producing a Δ*W*_peak_ value lower than the defined threshold (indicating no salivary responses). When ten −0.25 V pulse signals (at *T*_p_ = 0.5 s and Δ*t* = 0.5 s) were applied to the G1 electrode to simulate the “sight of food” (Figure 4b), the corresponding Δ*W*_peak_ value was higher than the threshold (indicating salivary responses).

The Δ*W*_peak_ value undergoes spontaneous attenuation, which drops rapidly in the initial phase and then gradually decreases. During training (Figure 4c), three −0.25 V and ten −0.25 V pulse signals were applied simultaneously to the G1 and G2 electrodes. After training (Figure 4d), even with only three −0.25 V pulse signal inputs (bell ringing), a Δ*W*_peak_ value greater than the threshold (indicating salivary response) is clearly produced. This behavior demonstrates an effective link between the input signals applied to the G1 and G2 electrodes, indicating that our highly interconnected multiterminal neural devices can learn to associate ten −0.25 V pulse signals with three −0.25 V pulse signals.

At high humidity conditions (Figure 4e–h), we set a threshold of −2.23 μA described by the orange dotted line to correspond to the suppression of appetite derived from IPSC within the CNC composite-EGT. First (Figure 4e), three −0.25 V pulse signals (at *T*_p_ = 0.5 s and Δ*t* = 2 s) were introduced to the G2 electrode to simulate the “bell ringing”, which generated a Δ*W*_peak_ value lower than the defined threshold, indicating no suppression responses. Ten −0.25 V pulse signals (at *T*_p_ = 0.5 s and Δ*t* = 0.5 s) were applied to the G1 electrode to simulate the “sight of food” (Figure 4f), which corresponds to the higher value of Δ*W*_peak_ than the threshold, indicating suppression responses. The training process corresponds to the simultaneous bias of three −0.25 and ten −0.25 V pulses to the G1 and G2 electrodes (Figure 4g). As a result of training (Figure 4h), only three signal inputs of −0.25 V, a higher value of Δ*W*_peak_ than the threshold value, were generated, indicating suppression response.

Figure S5 exhibits long-term potentiation and depression of the devices. By repeated pulses of stimulation to the system, the potentiation and depression characteristic curve of the synaptic transistors was measured. The same sequence of the 50 voltage pulses of −0.25 V (at *T*_p_ = 0.5 s and Δ*t* = 0.5 s) followed by 0.25 V (at *T*_p_ = 0.5 s and Δ*t* = 0.5 s) generated potentiation/depression and depression/potentiation sequences for low and high humidity conditions, respectively. During the potentiation and depression process, the controlled speed of ion migration within the electrolyte layer might tune the linear and symmetric synaptic weight change.^69,70^ To confirm the uniform electrical properties of the artificial neural networks, the cycle-to-cycle and device-to-device variations of the synaptic transistors were measured.^71^ It showed an averaged cycle-to-cycle variation of <5%, based on 20 repeated tests (Figure S7a). Moreover, the averaged device-to-device variation of 10 device measurements was found to be low, below 10% (Figure S7b).

### Synergy of Photonic and Electric Signals for Superhuman Intelligent Chirality Recognition

**Figure 5:**
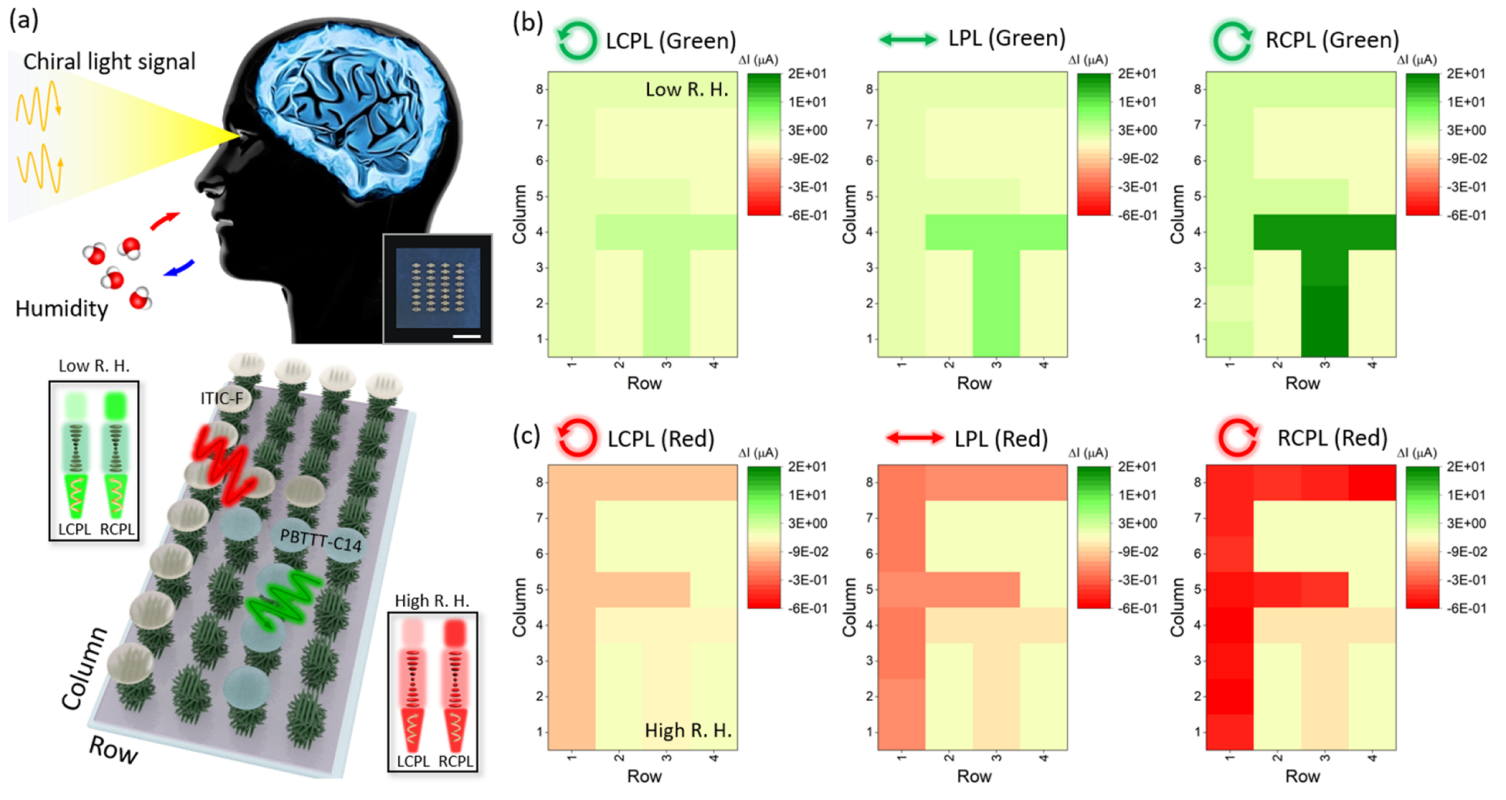
Superhuman visual system by facilitating chiral light detective CNC composite layer. (a) Schematically illustrated advanced visual system based on the actual 4 × 8 synaptic device arrays (inset scale bar: 1 cm). Note that the CNC composite layer transmits through the incident light differently depending on humidity and the polarization state. At low humidity conditions, the transmittance of green RCPL is higher than that of green LCPL. For high humidity conditions, the larger chiral pitch of CNC composites restricts the red LCPL’s transmittance compared to the red RCPL. Based on the inkjet-printed PBTTT-C14 and ITIC-F semiconducting channels, the English alphabet letters (b) T and (c) F are differently recognized within the system by modulating the wavelength and polarization state of the incident lights under electric pulses.

Figure 5a illustrates the advanced visual system of the artificial brain, perceiving external information through the eyes. The visual process involves the detection of light by the eye, extraction of color and chirality by the retina, and analysis of information by the central visual system. Unlike a simple light and dark pattern, the advanced retina separates and sends information about different colors and chirality to the occipital lobe of the central vision system. This enables the advanced artificial brain to accurately identify the letter with high recognition depending on humidity conditions. To demonstrate complex patterns with colors and chirality, 32 chiro-optoelectronic synapses were used to store and identify images made up of 4 × 8 pixel units (the inset image of Figure 5a).

PBTTT-C14 and ITIC-F organic semiconducting channels were inkjet printed (Figure S8) to create the capital letters T and F (Figure 5b and Figure S6). The different polarization states of light of 540 and 730 nm wavelengths were introduced to stimulate each pixel unit of the synaptic array in a specific order, recognizing each capital letter of T and F for low and high humidity conditions, respectively. Note that at low and high humidity conditions, CNC composite layers show green and red reflection colors, respectively, and the right-handed circularly polarized light (RCPL) of 540 and 730 nm wavelengths shows high transmittance through the layers compared to left-handed circularly polarized light (LCPL) (Figure S9).^54^ The transfer characteristics of PBTTT-C14 and ITIC-F synaptic transistors were analyzed depending on the polarization state of the incident light and humidity conditions (Figure S10).

The postsynaptic current response under different light irradiations was obtained 5 s after applying ten −0.25 V pulse signals (Figure S11). At first, the synaptic array could differentiate the letter T with the varied polarization state of green light, which is an advanced platform similar to how the eye of an insect sends information to the retina. As the transmittance of incident green light through the CNC composite layer increases in the order of LCPL, linearly polarized light (LPL), and RCPL, the subsequent optoelectronic signals of the PBTTT-C14 region increase. After 150 s of three −1 V electric pulses under different polarization states of green light, the degree of positive optoelectrical signals (Δ*I*) corresponding to the excitatory behavior was compared quantitively. The higher the value of positive Δ*I*, the more distinct the recognition of the letter T at low humidity conditions. For the inkjet-printed ITIC-F portion, the negative values of Δ*I* generated at high humidity conditions corresponds to the inhibitory behavior. Following the same sequence of the polarization state of the red light, LCPL, LPL, and RCPL, the vague recognition transits to the distinct letter F recognition. These results show that the advanced photonic synapses can recognize the positive or negative value of Δ*I* and chirality of optical signals as well as wavelengths, which is a unique feature compared with other existing photonic synapses that can only distinguish grayscale information. This innovative analyzing and recognizing ability is due to the modulation in the hysteresis behavior of EGT and selective transmittance and chirality of the incident light depending on humidity conditions.

The human visual system processes external information differently based on factors such as emotions and interests, leading to varying levels of memory retention, which corresponds to the humidity condition in the systems. By mimicking this complex behavior, the photonic synapse can selectively remember the information of interest. In Figure 5b,c, the letters T and F were recorded by a photonic synaptic array applying the same electrical pulses at different humidity conditions.

This varied concentration of water molecules corresponds to potentiated and depressed interest in the input image. The additional tuned chirality of the incident light is much clearer than the case of the unexposed condition, even though both were stimulated with the same amount of electrical inputs.

## Conclusion

We suggested advanced artificial synapses by deploying humidity-triggered optoelectronic properties to imitate excitatory and inhibitory functions. The degree of absorbed water molecules in the chiral electrolyte layers modulated the current of backward sweeping and the transmittance of the incident light, triggering excitatory or inhibitory postsynaptic behavior and the vague or distinct recognition of letters. The multifunctional CNC composite layer-based EGT showed tunable *I*–*V* characteristics and a wide range of various typical postsynaptic behaviors. By taking advantage of the humidity-responsive hysteresis properties, Pavlov’s learning rule exhibiting salivation and depression of appetite was simulated in multigate devices. Our BEGTs can further realize the detection of light information, the extraction of intensity and color information, and colored pattern recognition and memory. The dual synaptic plasticity can be demonstrated by adjusting the polarization state of the incident light, affording selective detection, processing, and memorization of the information of interest with excitatory and inhibitory behavior. In the human visual platform, the retina does not simply transmit information about the patterns of light and dark that fall on it. Instead, it extracts information about different colors and sends it to the occipital lobe of the central vision system.^72^ Beyond this, the suggested superhuman intelligence could detect light information by the eye and the extraction of color by the retina, combined with analysis of the information, simultaneously.

We expect that this study not only broadens the fundamental understanding of humidity-triggered optoelectronic properties for synaptic behavior but also suggests applications of high-density multilevel electronic signal regulation related to an emulation of chiral light-driven synaptic activity normal to the living systems of insects.^73−76^ The proposed multifunctional EGTs can be explored as a building block for complex image recognition with an artificial retina because they can detect the additional polarization states of the incident light. Future efforts will aim to make progress in neuromorphic material designs with large-scale programmable circuits for highly effective image recognition and categorization with simultaneous chirality detection. Furthermore, the multiple stimuli-triggered electronic signals of our device need to be elaborately validated for bionic eyes without complex artificial neural network processing. In an artificial visual platform, such synergy can be facilitated via tunable transmittance of the incident light depending on the polarization state and environmental adaption. The interdisciplinary techniques of chiro-optics, electronics, and biology establish that multifunctional electrolyte-based synaptic devices present great potential for the strategic development of next-generation bio-mimetic neuromorphic devices and neurorobotics applications.

## Methods/Experimental Section

### Fabrication of CNC-Complex Layer

To demonstrate a uniformly spread CNC complex solution onto a hydrophobic ITO/glass substrate, a UV/ozone treatment for 30 min was conducted (PSD Series, Digital UV Ozone System by NOVASCAN). Then, the substrate was attached to a round-shaped Petri dish (diameter: ∼60 mm) using double-sided tape. The prepared CNC complex solution was poured, forming the desired photonic film by an evaporation-induced self-assembly. The solvent evaporation process was proceeded for ∼3 days in a controlled environment (25% RH, *T* = 25 °C). Details of the CNC preparation are described in the Supporting Information.

### Device Fabrication Based on Inkjet-Printed OSC

The p- and n-types of OSC such as poly[2,5-bis(3-tetradecylthiophen-2-yl)thieno[3,2-*b*]thiophene] (PBTTT-C14) (Sigma-Aldrich) and 9-bis(2-methylene-((3-(1,1-dicyanomethylene)-6,7-difluoro)indanone))-5,5,11,11-tetrakis(4-hexylphenyl)-dithieno[2,3-*d*:2′,3′-*d*′]-*s*-indaceno[1,2-*b*:5,6-*b*′]dithiophene (ITIC-F) (Sigma-Aldrich), respectively, were prepared with a concentration of 5 mg/mL in chlorobenzene (Sigma-Aldrich). The active channel area was ∼500 × 50 μm^2^ by controlling the velocity of the scanning operation and the burst number of the droplets. Lastly, a 50 nm thickness of an Au source/drain electrode was deposited on top of the OSC through thermal evaporation via a shadow mask under 5.0 × 10^–6^ Torr, whose channel length (*L*) and width (*W*) were 500 and 50 μm, respectively. All of the detailed optoelectronic characterizations of the CNC composite-layer-based EGT are presented in the Supporting Information.

## Supporting Information Available

Experimental details and additional data as noted in the text such as microscopy analysis of CNC composite layers, electrical characteristics of EGTs, topological and spectroscopy analysis of semiconductor films, and optical characteristics of CNC composite layers depending on humidity conditions (PDF)
