# Supplementary material for: Trainable Bilingual Synaptic Functions in Bio-enabled Synaptic Transistors
Source: ACS Nano. 2023 Sep 18;17(19):18883–92. doi: 10.1021/acsnano.3c04113 (PMC10569090; doi:10.1021/acsnano.3c04113)
Supplement: Supplementary file 1 — nn3c04113_si_001.pdf [file nn3c04113_si_001.pdf]

Supplementary Information

Trainable Bilingual Synaptic Functions in Bio-enabled Synaptic Transistors

Moon Jong Han[1] *and Vladimir V. Tsukruk*[2,*]

[1]*Department of Electronic Engineering, Gachon University, Seongnam, 13120, Republic of Korea*

[2]*School of Materials Science and Engineering, Georgia Institute of Technology, Atlanta, GA 30332, USA*

*To whom correspondence should be addressed.

E-mail: Vladimir@mse.gatech.edu

Table of Contents

Experimental section

Figure S1. Microscopy analysis of CNC composite layer**.**

Figure S2. The transfer characteristics of PBTTT-C14 and ITIC-F-based EGTs**.**

Figure S3. The pronounced electrical hysteresis curves depending on gate sweeping rates.

Figure S4. PPF and PPD as a function of interval times.

Figure S5. Spike voltage-dependent plasticity (SVDP) of the synaptic device**.**

Figure S6. Retention characterization of the CNC composite-gated transistors.

Figure S7. Potentiation/depression characteristic curves of the synaptic transistors.

Figure S8. Topological and UV-Vis spectroscopy analysis of ink-jet printed PBTTT-C14 and ITIC-F films.

Figure S9. Transmittance and CD spectra measurement of CNC composite layer depending on humidity**.**

Figure S10. Transfer characteristics of PBTTT-C14 and ITIC-F transistors.

Figure S11. The PSCs of PBTTT-C14 and ITIC-F synaptic transistors.

Experimental section

CNC Preparation:

CNCs were synthesized by using wood pulp through the sulfuric acid hydrolysis method.[1] The dried wood pulp in 64 wt% sulfuric acids was continuously stirred at 45 ºC. The hydrolysis was performed for 60 minutes, and the reaction was quenched by the addition of a tenfold volume of MiliQ Nanopure water to the 64% sulfuric acid solution. The diluted solution was kept at 24 ºC for 12 hours. The clear supernatant was removed, and the precipitate was washed by using centrifugation with MiliQ Nanopure water. The washed solution in a dialysis tube (14kDa molecular weight cut off) was dialyzed against deionized distilled water until the pH value of deionized water became constant (~ 72 hours). The deionized water was replaced every 3 hours during the dialysis. The resulting solution was centrifuged twice at 11,000 rpm. The collected supernatant was then sonicated by a large tip-sonicator (1.2 cm diameter probe, Q700 model, QSonica) for 4 minutes and 30 seconds (5 seconds on and 5 seconds off and 40% amplitude).

Preparation of CNC/PEG/NaCl Nanocomposite Films:

3 mL from 1 wt % PEG (M_w = 2 kDa) (Sigma Aldrich) aqueous solution was added to the 10 mL 1 wt % CNC suspension with magnetic stirring for 2 h at room temperature. Then, 100 µL from 0.5 wt % NaCl (Sigma Aldrich) aqueous solution was further introduced to the CNC/PEG solution followed by stirring for 2h at room temperature.

Fabrication of CNC-complex layer:

To demonstrate uniformly spread CNC complex solution onto the hydrophobic ITO/glass substrate, a UV/Ozone treatment for 30 min was conducted (PSD Series, Digital UV Ozone System by NOVASCAN). Then, the substrate was attached to the round-shaped Petri dish (diameter: ~ 60 mm) using double-sided tape. The prepared CNC complex solution was poured, forming the desired photonic film by evaporation-induced self-assembly. The solvent evaporation process was undergone for ~ 3 days in a controlled environment (25% RH, T = 25 °C).

Humidity control of CNC-complex film:

The humidity control of CNC-complex film at different RH was conducted by placing the film in humidity chambers with the desired RH. The 42% RH, 67% RH, 92% RH, and 97% RH (measured using a Cole-

Parmer hygrometer) were conditioned by using a saturated solution of potassium carbonate (K_2CO_3), potassium iodide (KI), potassium nitrate (KNO_3), and copper(II) sulfate pentahydrate ($CuSO_4 \cdot 5H_2O$), respectively.[2] The saturated solution-filled polystyrene (2.5 cm x 2.5 cm x 2.5 cm, Ted Pella) was located in borosilicate glass (8 inch x 6.5 inch x 3 inch, MICRO) sealed with a parafilm followed by a snap lid. To maintain the RH within the EGTs, the prepared top glass substrate was placed onto the bottom device substrate.

Device fabrication based on inkjet printed-OSC:

The poly[2,5-bis(3-tetradecylthiophen-2-yl)thieno[3,2-*b*]thiophene] (PBTTT-C14) (Sigma Aldrich) and 9-Bis(2-methylene-((3-(1,1-dicyanomethylene)-6,7-difluoro)-indanone))-5,5,11,11-tetrakis(4-hexylphenyl)-dithieno [2,3-d:2',3'-d']-s-indaceno[1,2-*b*:5,6-*b*']dithiophene (ITIC-F) (Sigma Aldrich) were dissolved with a concentration of 5 mg/ mL in chlorobenzene (Sigma Aldrich). The inkjet processing was accomplished by a Microfab JETLAB II with a 40 μm diameter piezoelectric-driven inkjet nozzle with a motorized stage showing an accuracy of 5 μm. The active channel area was ~ 500 × 50 μm^2 by applying the optimized velocity of scanning operation and the burst number of the droplets corresponding to 1 cm/s and 50, respectively, onto the prepared CNC complex/ITO/glass substrates. Subsequently, the 50 nm-thickness of Au source/drain electrodes was patterned on top of the OSC through thermal evaporation via a shadow mask under 5.0×10^{-6} Torr, whose channel length (L) and width (W) 500 and 50 μm, respectively.

Optoelectrical characterization:

The capacitance of the CNC complex layers as a function of frequency under different humidity was determined by impedance spectroscopy using an LCR meter (4287A, Agilent) in a frequency range of 10 mHz to 0.1 MHz with an AC excitation voltage of 500 mV, based on Au/CNC-complex/ITO metal/dielectric/metal layer structures. The EGTs were characterized at RT by using a semiconductor parameter analyzer (4200-SCS, Keithley). The transfer and output curves were obtained and charge carrier mobilities were estimated by the equation under the saturation regime;

$$I_{DS} = \left(\frac{WC_i}{2L}\right)\mu(V_{GS} - V_{th})^2$$

where C_i is the geometric capacitance of the CNC-complex at each RH condition. The threshold voltage (V_{th}) was estimated by a linear fitting in the linear transfer characteristics.

The optoelectrical characterization was performed on the dark and under LED (Thorlabs) illumination conditions (~ 1 mW cm^{-2}), whose electroluminescence emission peaks were ~ 550 and 730 nm. Before

the optoelectrical experiments, the distinct exposed optical powers were evaluated by a Thorlabs 120 UV power sensor. Different handed CPL exposure were conducted by the experimental sequence of LCPL and RCPL by a rotating linear polarizer, where the rotation of the polarizer 45° clockwise from the crossed polarizer and the quarter-wave plate transmitted the RCPL and vice versa.

UV-vis spectroscopy:

Light reflection characteristic of the samples was measured by using a Shimadzu UV-3600 spectrometer. For the light reflection characteristic measurement of the films depending on RH, freely standing films were placed between quartz slides and then conditioned in the humidity control chamber for 96 hours. In order to analyze the response of the films to the circularly polarized light, the transmission was measured under a circular polarizer.

CD spectroscopy:

CD spectra were collected by utilizing an Applied Photophysics Chirascan™-plus with the samples positioned perpendicularly to the beam path in a wavelength range between 300 and 800 nm.

Materials characterization:

The AFM tapping mode images were collected on the ICON Dimension microscope (Bruker) according to the usual routine[3]. The scan rate and resolution of the measurement were in the range of ～0.8 Hz and 512 × 512 pixels, respectively. AFM tips (MikroMasch, HQ:XSC11/AL BS) with a tip radius and a spring constant of ～10 nm and ～2.0 N m^{-1}, respectively, were used.

SEM measurement was conducted by a Hitachi SU-8230 instrument, applying 5 kV accelerating voltage. The cross-section image of the CNC complex film was obtained by cutting with a razor blade and adhering the film piece with carbon tape onto the mount side. The gold sputter coating was performed on the sample with gold for ~ 60 s, for a layer thickness of about 2 nm.

Polarized optical microscope measurement of CNC complex film was performed with an Olympus BX51 microscope under the reflection mode to characterize the photonic structures.

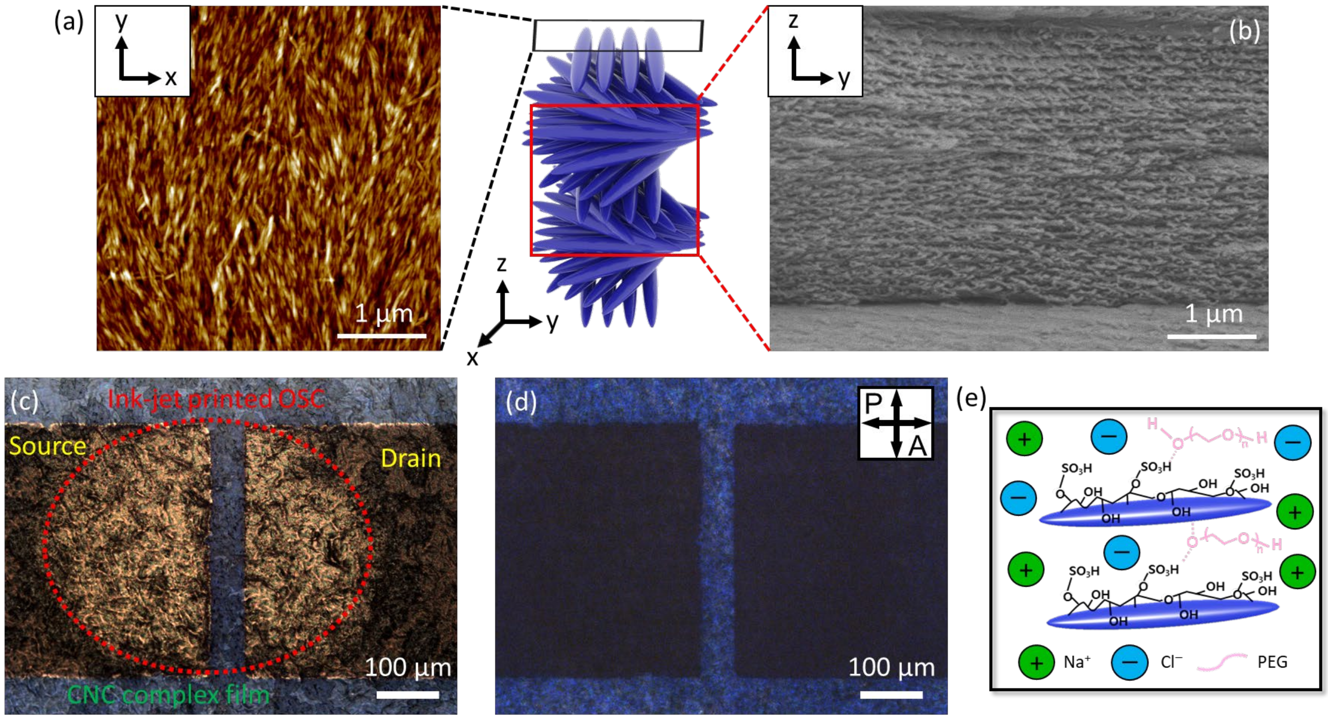

Figure S1. Microscopy analysis of CNC composite layer. (a) AFM and (b) SEM measurement technique was used to analyze the chiral nematic configuration of the CNC composite layer. (c-d) Optical and polarized optical microscopy image of the individual synaptic electrolyte-gated transistor based on ink-jet printed OSC. The semiconducting channel width and length are 500 and 50 μm, respectively. In the POM image, the formed chiral photonic structure of the CNC composite (CNC : PEG : NaCl) layer is confirmed. (e) Schematic illustration of CNC : PEG : NaCl composite film after evaporation-induced self-assembly.

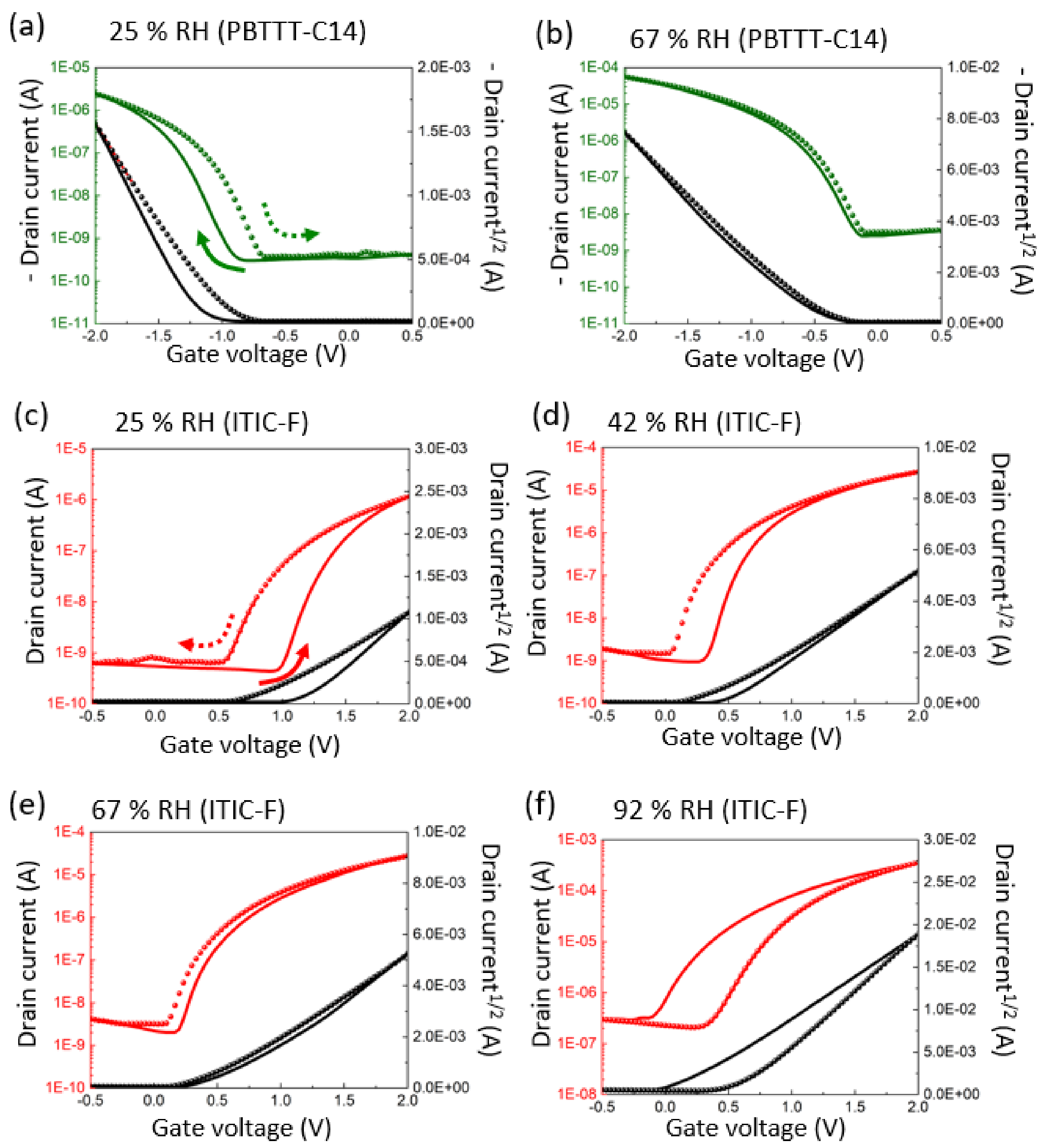

Figure S2. The transfer characteristics of PBTTT-C14 and ITIC-F-based EGTs measured by the dual sweeping of the gate voltage at V_{DS} = -2V under different relative humidity conditions.

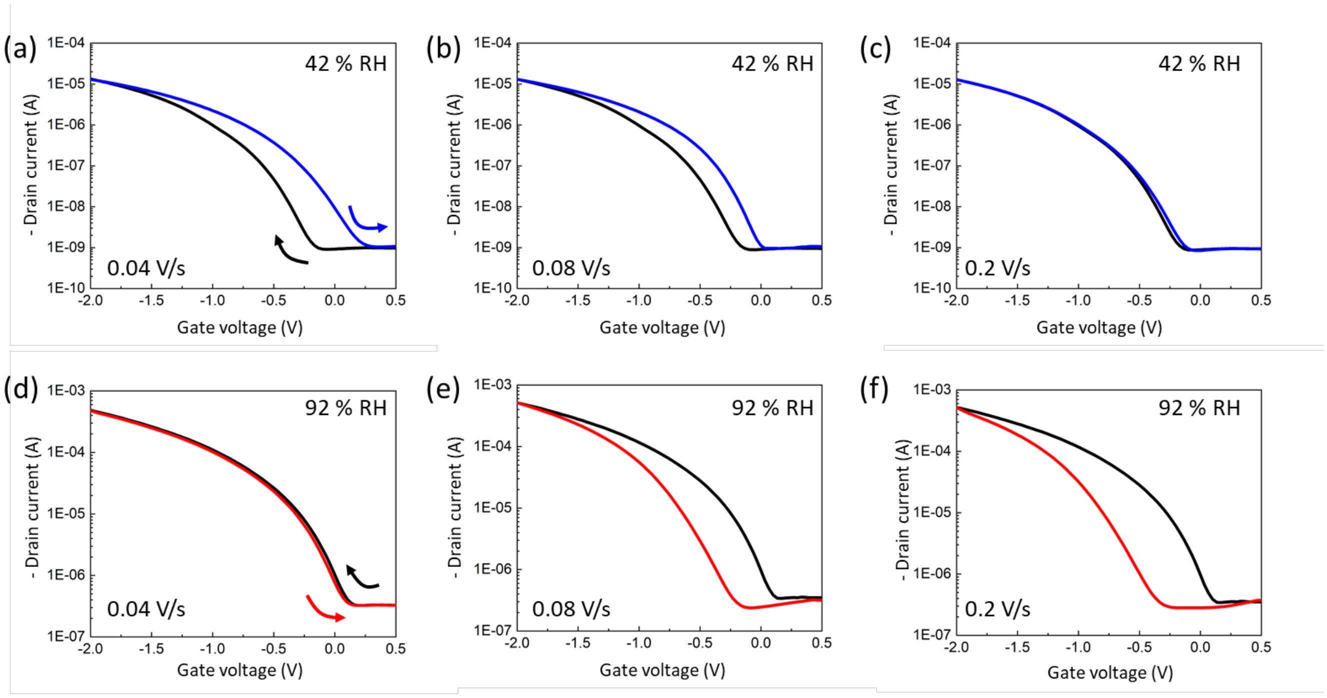

Figure S3. The pronounced electrical hysteresis curves depending on gate sweeping rates. Under low humidity conditions (a-c), the clockwise hysteresis is pronounced at a lower sweeping rate, driven by the dominant capacitive gating effect. For high humidity conditions (d-f), the anticlockwise hysteresis is pronounced at a higher sweeping rate due to the trapped charges by the water molecules within the CNC composite/semiconductor layers.

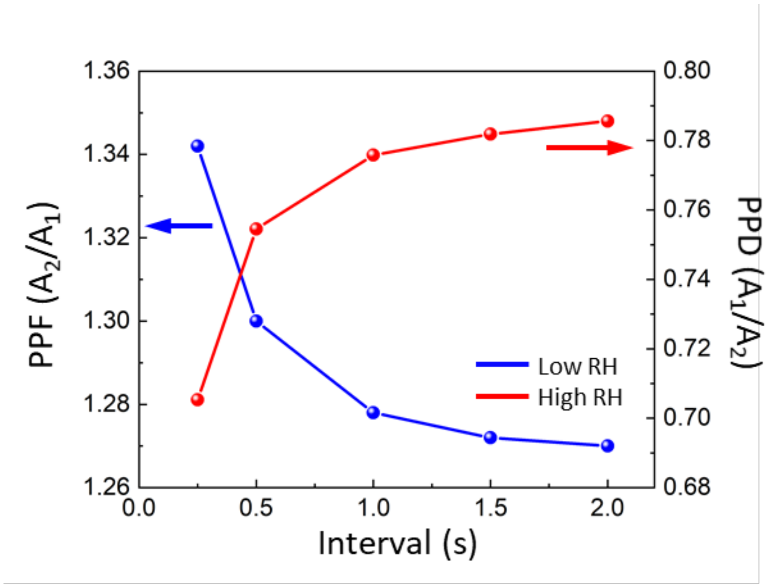

Figure S4. PPF and PPD as a function of interval times such as 0.25, 0.5, 1, 1.5, and 2 s for low and high humidity conditions.

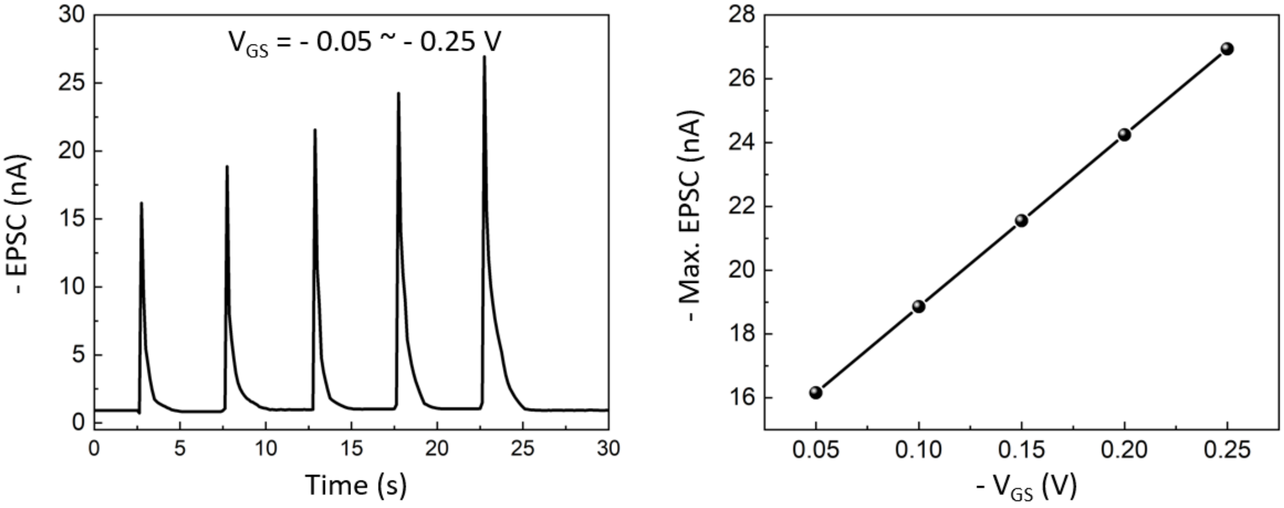

Figure S5. Spike voltage-dependent plasticity (SVDP) of the synaptic device with various gate voltages from −0.05 to −0.25 V.

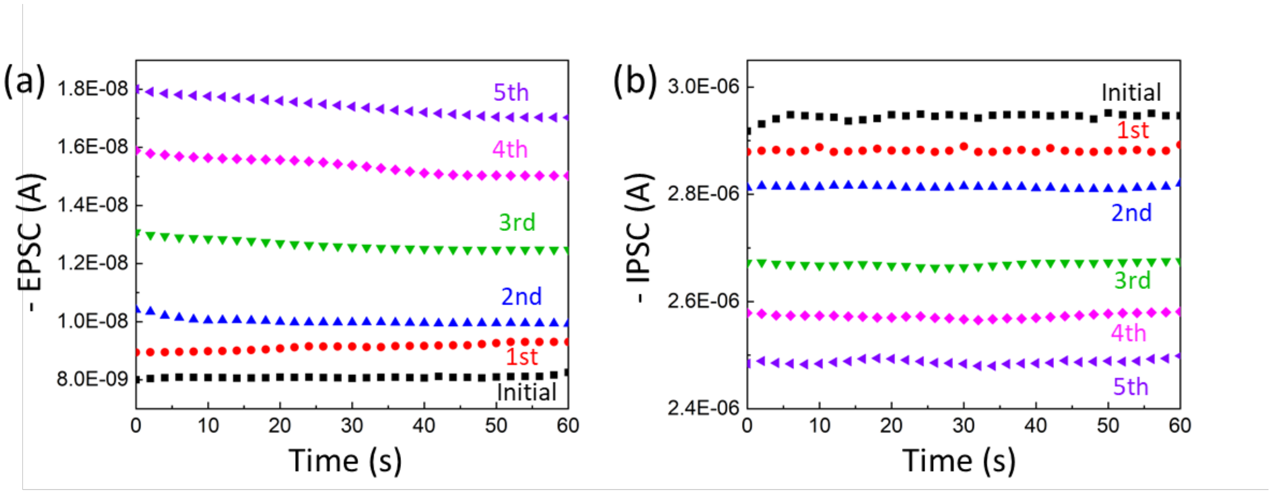

Figure S6. Retention characterization of the CNC composite-gated transistors of the initial state and after five consecutive V_G sweeps (0 V → – 0.25 V → 0 V) under (a) low and (b) high humidity conditions. They showed an increase and decrease in the distinguishable states for 60 s.

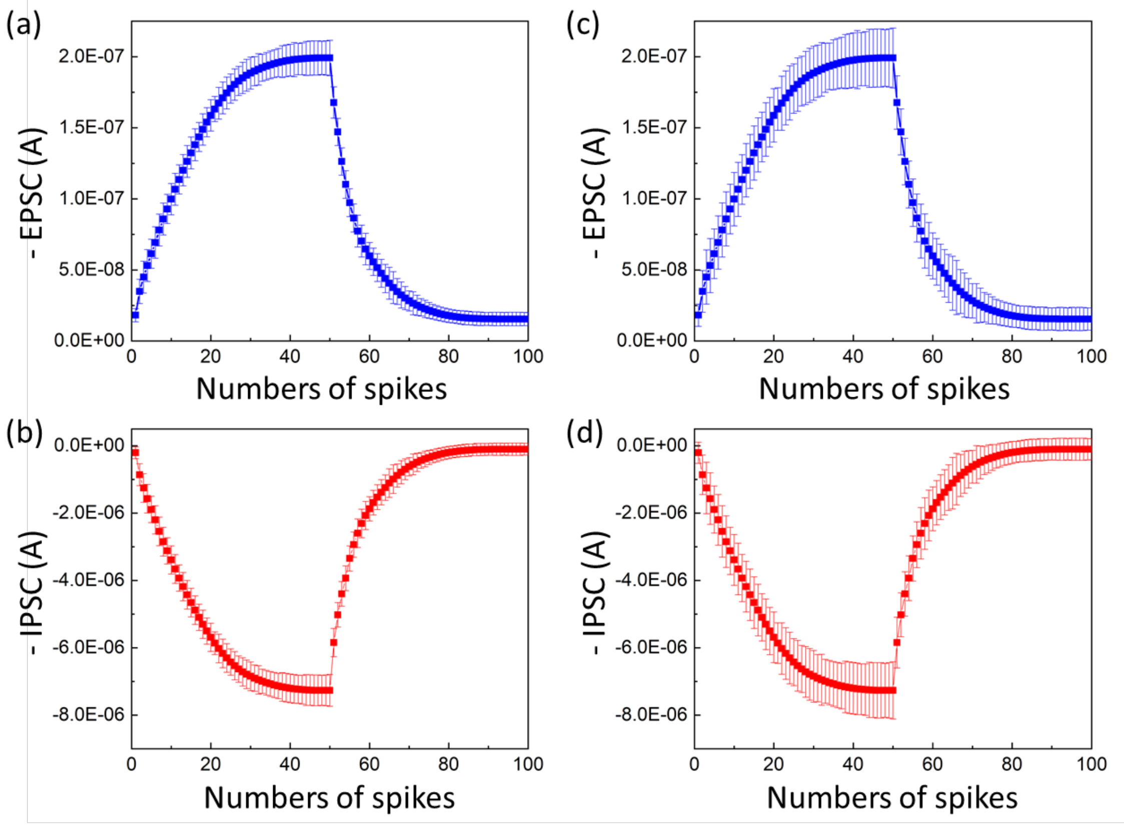

Figure S7. Potentiation/depression characteristic curves of the synaptic transistors. Cycle-to-cycle variation of (a) low and (b) high humidity conditions in 20 pulse tests. The same sequence of the 50 voltage pulses of − 0.25 V (at Tp = 0.5 s and Δt = 0.5 s) followed by 0.25 V (at Tp = 0.5 s and Δt = 0.5 s) generated potentiation/depression and depression/potentiation sequence for low and high humidity conditions, respectively. The average cycle-to-cycle variation is <5%. Device-to-device variation of (c) low and (d) high humidity conditions, based on 10 synaptic transistors. The average device-to-device variation is <10%.

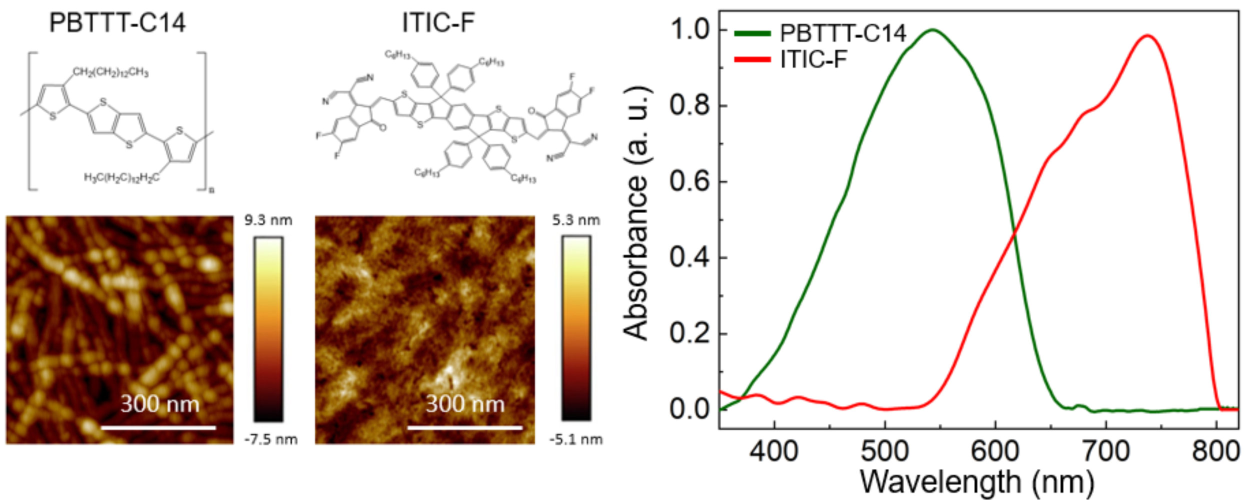

Figure S8. Topological and UV-Vis spectroscopy analysis of ink-jet printed PBTTT-C14 and ITIC-F films.

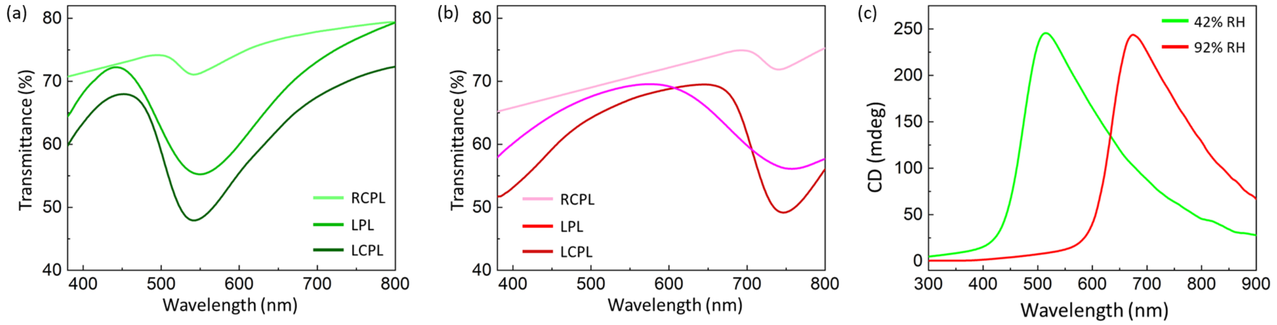

Figure S9. Transmittance measurement of CNC composite layer depending on humidity such as (a) 42 and (b) 92 RH % and CD spectroscopy measurement.[4]

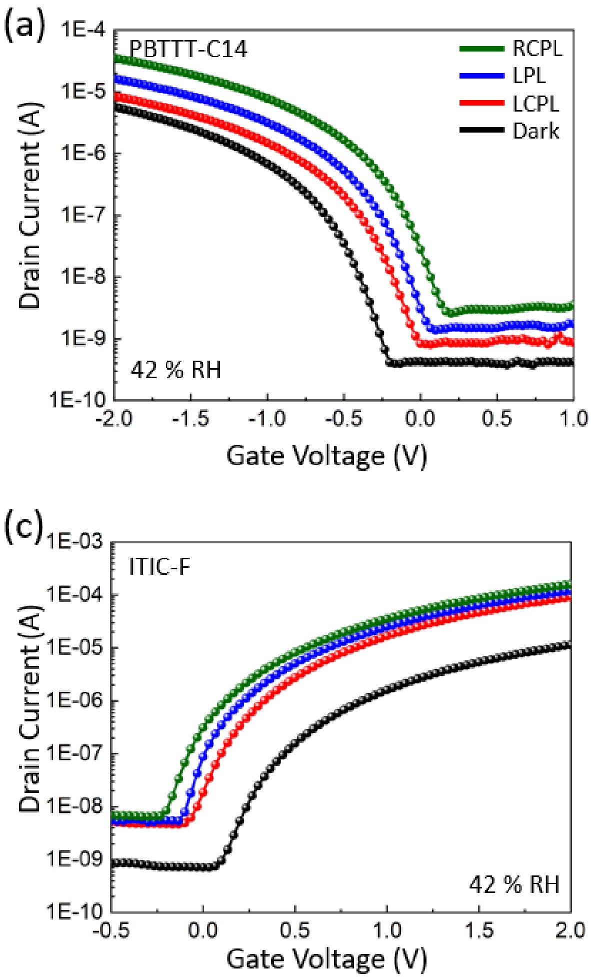

Figure S10. Transfer characteristics of (a,b) PBTTT-C14 and (c,d) ITIC-F transistors at (a,c) 42 % RH and (b,d) 92 % RH conditions.

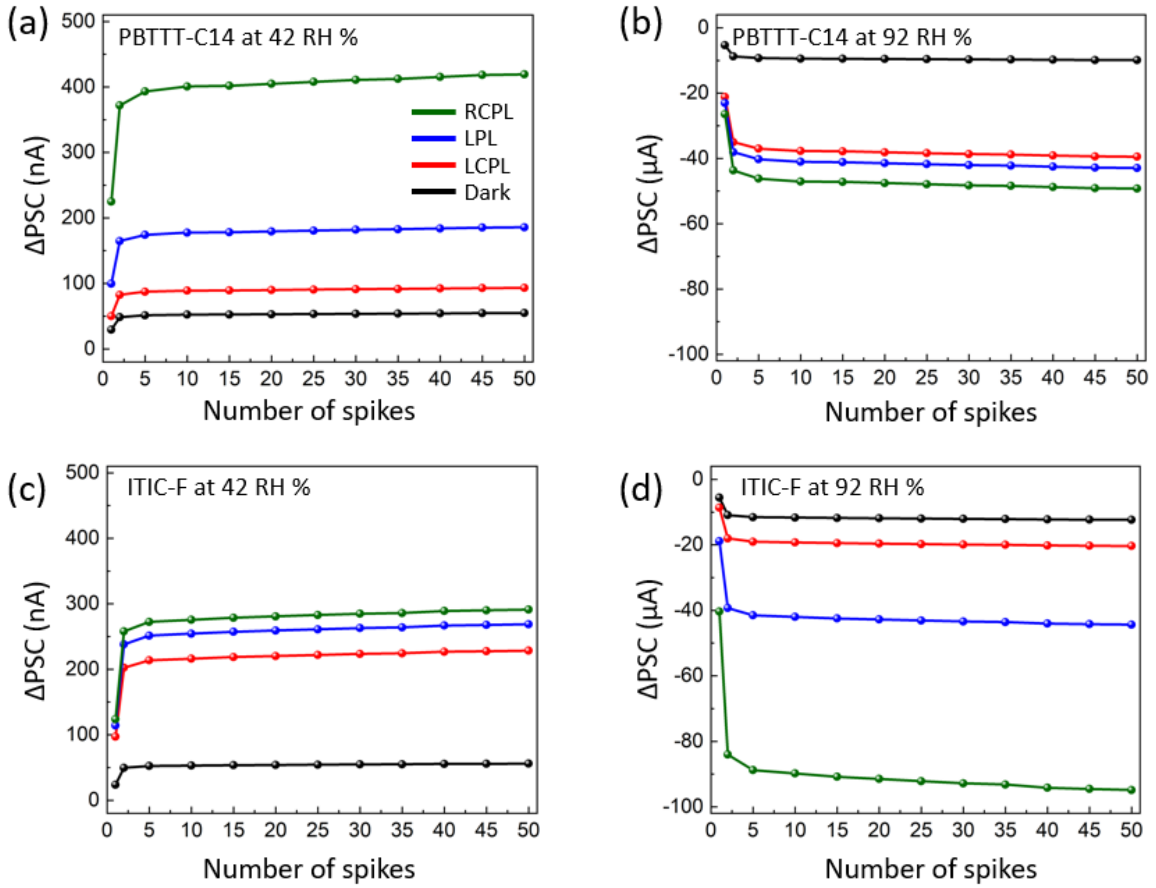

Figure S11. The PSCs of (a,b) PBTTT-C14 and (c,d) ITIC-F synaptic transistors under 50 consecutive electric pulses under different polarization states of light such as dark, left-handedness circularly polarized light (LCPL), linearly polarized light (LPL), and right-handedness circularly polarized light (RCPL) at varied humidity conditions such as (a,c) 42 and (b,d) 92 RH %.

[1] Beck-Candanedo, S., Roman, M. & Gray, D. G. Effect of Reaction Conditions on the Properties and Behavior of Wood Cellulose Nanocrystal Suspensions. *Biomacromolecules* **6**, 1048–1054 (2005).

[2] Greenspan, L. Humidity fixed-points of binary saturated aqueous-solutions. *J. Res. Natl Bur. Stand. Sect. A* **81**, 89–96 (1977).

[3] McConney, M. E., Singamaneni, S. & Tsukruk, V. V. Probing Soft Matter with the Atomic Force Microscopies: Imaging and Force Spectroscopy. *Polymer Reviews* **50**, 235–286 (2010).

[4] Han, M. J.; Kim, M.; Tsukruk, V. V. Chiro-Optoelectronic Encodable Multilevel Thin Film Electronic Elements with Active Bio-Organic Electrolyte Layer. *Small* **2023**,*19* (18), 2207921.
